# Soft, Skin-Conformal Electronic Interfaces for Multimodal Biosignal Monitoring and Transcutaneous Stimulation

**DOI:** 10.1021/acsami.6c02518

**Published:** 2026-04-28

**Authors:** Md Saifur Rahman, Ziyu Zhu, Nicholas B. Abadie, Kailash Pillai, Adam Reyes, Luke McKinley, Creed G. Bielss, Salina Teng, Jie Zhao, Dimitry G. Sayenko, Limei Tian

**Affiliations:** 1 Department of Biomedical Engineering, Center for Remote Health Technologies and Systems, 14736 Texas A&M University, College Station, Texas 77843, United States; 2 Department of Electrical and Computer Engineering, 14736 Texas A&M University, College Station, Texas 77843, United States; 3 Department of Neurosurgery, Center for Neuroregeneration, Houston Methodist Research Institute, Houston, Texas 77030, United States

**Keywords:** PEDOT:PSS, conductive nanocomposite, soft electronics, ionic−electronic conduction, electrophysiological recording, transcutaneous electrical stimulation

## Abstract

Soft, stable, and high-performance skin-electrode interfaces are essential for continuous electrophysiological recording and transcutaneous electrical stimulation. Conventional gel electrodes suffer from dehydration, unstable skin-electrode contact, reduced recording quality, and limited stimulation efficiency during prolonged use. This paper reports a soft, mixed-conducting nanocomposite electrode composed of poly­(3,4-ethylenedioxythiophene):poly­(styrenesulfonate) (PEDOT:PSS) integrated with hygroscopic and ionic dopants. The synergistic formulation enhances mixed ionic–electronic conduction, mechanical softness, and long-term hydration stability. A simple micromolding process enables scalable fabrication of conformal, freestanding electrodes that adhere seamlessly to the skin. The optimized composition achieves an excellent balance between conductivity and softness, exhibiting approximately a 20-fold lower interfacial impedance and a 2.6-fold higher charge injection capacity compared to gel electrodes. As a result, these nanocomposite electrodes deliver higher signal-to-noise ratios in electrocardiography and electromyography recordings and enhanced bioimpedance sensitivity and achieve a 2-fold expansion of the stimulation window. This nanocomposite design establishes a versatile materials platform for soft, durable, and high-fidelity bioelectronic interfaces, enabling advances in wearable sensing and neuromodulation technologies.

## Graphical abstract

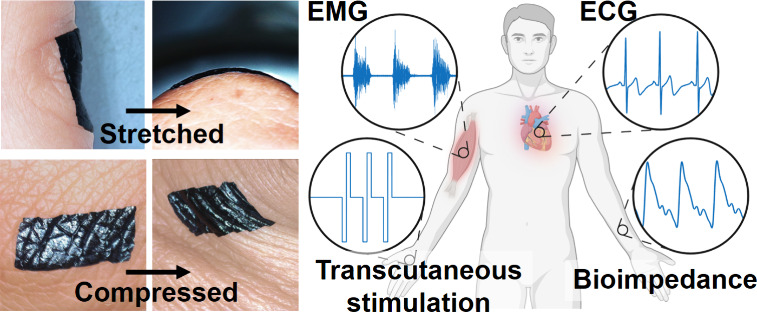

## Introduction

Wearable electronic interfaces that record and modulate electrical activity are fundamental tools in neuroscience, clinical monitoring, rehabilitation, and human-machine interface.^1,2^ The human body constantly generates a variety of biosignals, including the electrical activity of the heart, muscles, and brain, as well as hemodynamic signals associated with vascular function. Daily monitoring of these signals has become increasingly prevalent in both hospitals and home settings, supporting early diagnosis, personalized treatment, and improved health management.^3^ Among these modalities, electrocardiography (ECG), electromyography (EMG), and electroencephalography (EEG) provide critical insights into cardiovascular, neuromuscular, and neurological conditions.^4–6^ Bioimpedance measurements offer valuable information on tissue composition, hydration status, and physiological function, facilitating applications in disease assessment and body composition analysis.^7,8^ In addition to signal acquisition, skin-mounted electronic interfaces are being actively developed for electrical stimulation and feedback integration within closed-loop human-machine systems, enabling adaptive motor control and rehabilitation.^9,10^ The performance of these technologies depends critically on the electrode-skin interface, which governs both signal fidelity during recording and charge delivery efficiency during stimulation. Currently, most clinical and consumer systems rely on gel-based Ag/AgCl electrodes. While effective for short-term use, these electrodes suffer from progressive dehydration, unstable skin-electrode impedance, potential skin irritation, and limited durability. Such issues degrade signal quality, reduce stimulation efficiency, and ultimately constrain their use in wearable continuous operation.

Soft, stretchable conductive materials have emerged as a promising solution to maintain stable, low-impedance, and conformal contact with skin over extended periods.^11–14^ Ultrathin metal nanomeshes and conductive polymers have been investigated in this context, each offering distinct advantages.^15–22^ Among various conductive polymers, poly­(3,4-ethylenedioxythiophene):poly­(styrenesulfonate) (PEDOT:PSS), has attracted particular interest due to its high electrical conductivity, aqueous processability, and biocompatibility.^23,24^ Its mixed ionic-electronic conduction enables efficient charge transfer across the bioelectronic interface, which is particularly advantageous for electrophysiological recording.^25–28^ While these electrodes have demonstrated superior recording performance, mechanical compliance, and long-term skin compatibility, further material innovations and interfacial design strategies are needed to enhance the efficiency of transcutaneous stimulation.

Here, we present a soft, stretchable PEDOT:PSS-based nanocomposite engineered to provide a stable, low-impedance, and efficient interface for electrophysiological recording and transcutaneous stimulation. The nanocomposite incorporates d-sorbitol, glycerol, and potassium chloride (KCl) as multifunctional additives to enhance mixed ionic-electronic conduction, mechanical softness, and interfacial stability. A simple micromolding process yields freestanding, conformal electrodes compatible with diverse skin-mounted and wearable configurations. The optimized formulation achieves an excellent balance between electrical conductivity and softness, resulting in a ∼20-fold reduction in skin-electrode impedance and a 2.6-fold increase in charge-injection capacity compared to commercial Ag/AgCl gel electrodes. These properties allow the nanocomposite electrodes to deliver higher signal-to-noise ratios in ECG and EMG recordings, enhanced bioimpedance sensitivity, and superior stimulation efficiency and comfort compared to gel electrodes. These results demonstrate that the synergistic incorporation of hygroscopic and ionic dopants in PEDOT:PSS enables a soft, durable, and high-performance interface for long-term bioelectronic applications.

## Results and Discussion

### Design and Fabrication of Nanocomposites

The nanocomposite comprises PEDOT:PSS, d-sorbitol, glycerol, and KCl. (Figure 1a). d-Sorbitol promotes phase separation between PEDOT-rich and PSS-rich domains, leading to the formation of PEDOT-rich nanofibrils and improved electrical conductivity.^27^ Glycerol facilitates ionic mobility and hydration stability. KCl serves as an ionic dopant, providing additional ion conduction pathways that enable efficient mixed ionic-electronic transport within the composite. To fabricate nanocomposite electrodes, we employed a simple, low-cost micromolding approach to define electrode dimensions and pattern (Figure 1b). A PEDOT:PSS precursor solution containing additives and KCl filled a parafilm mold with the desired patterns, followed by controlled drying at 60 °C for 30 min and annealing at 130 °C for 30 min. High-temperature annealing facilitates physical cross-linking of PEDOT-rich nanofibrils, yielding a mechanically stable nanocomposite film. After annealing, a freestanding nanocomposite electrode can be easily released from the PDMS surface due to the presence of additives. Although the electrode undergoes drying and annealing, the hygroscopic additives and hydrated polymer network enable KCl to dissociate into K^+^ and Cl^–^ ions, which facilitate ionic conduction and charge transport at the electrode-skin interface. The nanocomposite electrodes can be transferred and assembled onto various soft and stretchable substrates, including medical-grade adhesives, for various wearable applications. These applications include continuous recording of ECG, EMG, and bioimpedance signals, and transcutaneous electrical stimulation (Figure 1c). Figure 1d shows optical images of nanocomposite films laminated onto human skin at various locations. The soft, stretchable nanocomposite follows the skin deformation without compromising the seamless contact. The seamless skin-electrode interface is important for high-quality biosignal recording and effective stimulation.

**Figure 1:**
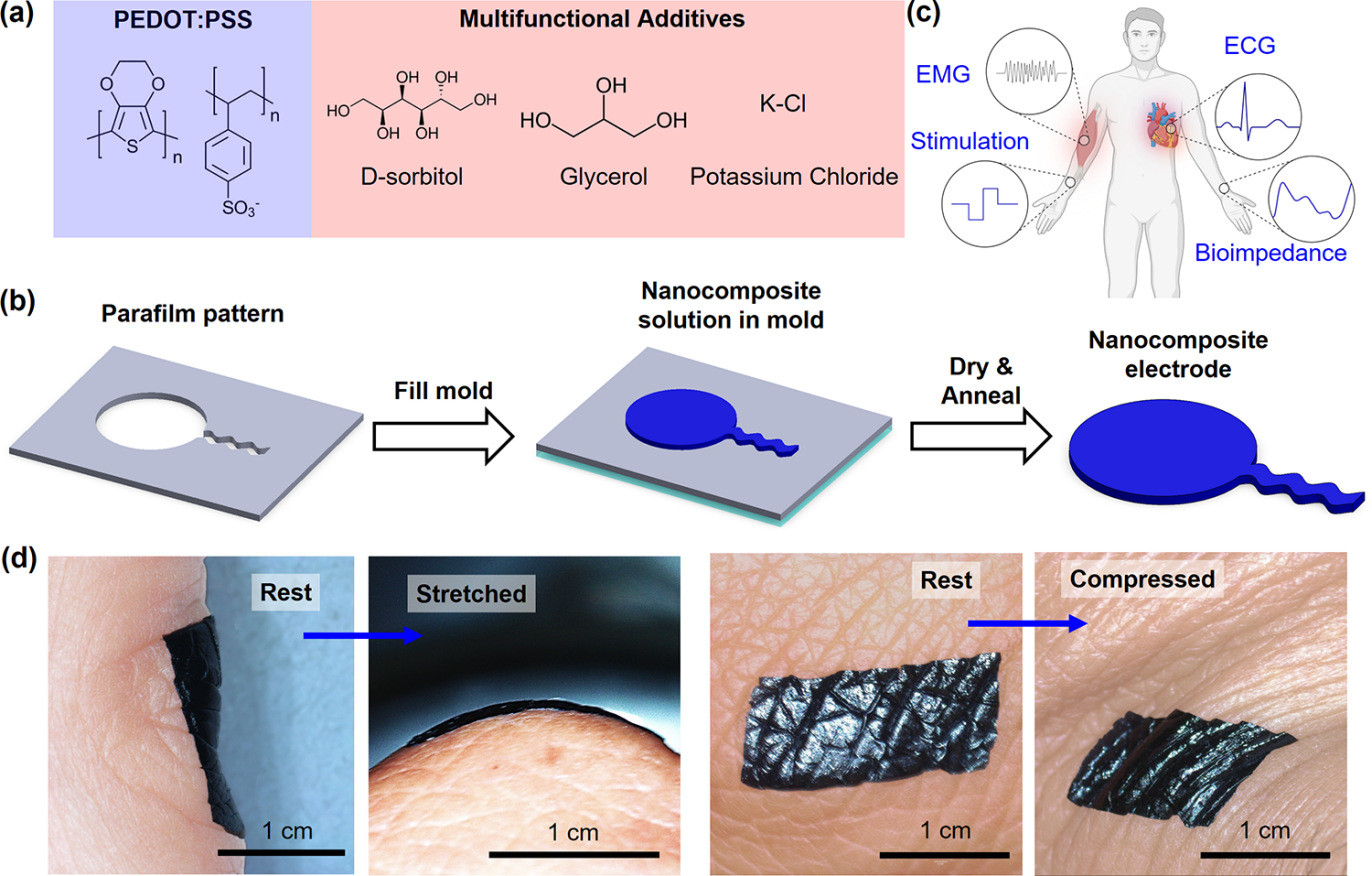
Synthesis and fabrication of skin-like nanocomposite electrodes. (a) Illustrations of conductive nanocomposite components. (b) Schematic illustration of the fabrication process. (c) Schematic illustration of multimodal sensing and transcutaneous stimulation applications. (d) Optical images showing the conformability of nanocomposite films on skin before and after stretch and compression.

### Nanocomposite Electrode Characterization

To evaluate the effect of ionic concentration on the nanocomposite properties, we systematically varied KCl concentration from 0.1 wt % to 0.4 wt % in the nanocomposite solution and characterized the mechanical and electrical properties of the resultant films. Tensile stress–strain measurements determine the Young’s modulus and stretchability of freestanding nanocomposite thin films with different KCl concentrations (Figure 2a). With increasing KCl concentration, the Young’s modulus gradually increased from ∼5 MPa at 0% KCl to ∼8 MPa at 0.4% KCl (Figure 2b). This trend shows that ionic loading reinforces the polymer network and increases the tensile modulus while keeping the modulus in the low MPa range for conformal skin contact. The tensile strength of the nanocomposite films ranges from 1 to 2 MPa across all formulations. The mechanical behavior also demonstrates a trade-off between Young’s modulus and stretchability, with higher stiffness corresponding to reduced elongation at fracture. The nanocomposite films with 0.1%–0.25% KCl sustained elongations over 30% before fracture, confirming their intrinsic, skin-like stretchability of ∼30% (Figure 2a).^16^ The stretchability allows the nanocomposite electrodes to conform to soft, dynamic tissue surfaces without mechanical failure. To evaluate electrical stability under stretch, we measured changes in resistance under 30% uniaxial strain to assess the effect of deformation on charge transport (Figure 2c). The resistance decreased from 2.3 Ω to 1.3 Ω as KCl increased from 0% to 0.25%. A further increase in KCl concentration to 0.4% resulted in a higher resistance. The change in resistance is less than 4% with a 30% stretch, confirming electrical stability. These results demonstrate that 0.25% KCl significantly reduces nanocomposite resistance by enhancing mixed ionic-electronic transport while maintaining a low modulus and high stretchability for the skin interface. High ionic loading (0.4% KCl) during synthesis may disrupt the percolated PEDOT conductive network, leading to reduced electronic conductivity and increased heterogeneity within the composite. To further elucidate the roles of d-sorbitol and glycerol, we prepared nanocomposites without each additive while maintaining the optimized KCl concentration (0.25 wt %) and measured the resulting mechanical and electrical properties. The nanocomposites exhibited a tensile modulus of ∼55 MPa without d-sorbitol and ∼16 MPa without glycerol, both substantially higher than those measured for samples without KCl (∼5 MPa) and with 0.25 wt % KCl (∼7 MPa) (Figure S1a,b). These results indicate that d-sorbitol and glycerol synergistically reduce the modulus of the nanocomposite, with d-sorbitol contributing more than glycerol to enhanced stretchability. In contrast, KCl slightly increases the modulus and reduces the stretchability. In addition, d-sorbitol, glycerol, and KCl collectively enhance the electrical conductivity of the nanocomposite (Figure S1c). We further characterized the microscopic and molecular structure of the nanocomposite with and without KCl. Scanning electron microscopy (SEM) images of the nanocomposite cross sections revealed a layered morphology resulting from the drying process, with no noticeable differences between samples with and without KCl (Figure S2). Raman spectra showed that the C_α_=C_β_ stretching vibration of the thiophene rings in PEDOT shifted from 1425 to 1417 cm^–1^ upon KCl addition (Figure 2d). This shift indicates PEDOT’s transition from a coiled to a more expanded or linear conformation, which enhances interchain charge transport and reduces sheet resistance.^27,29^

**Figure 2:**
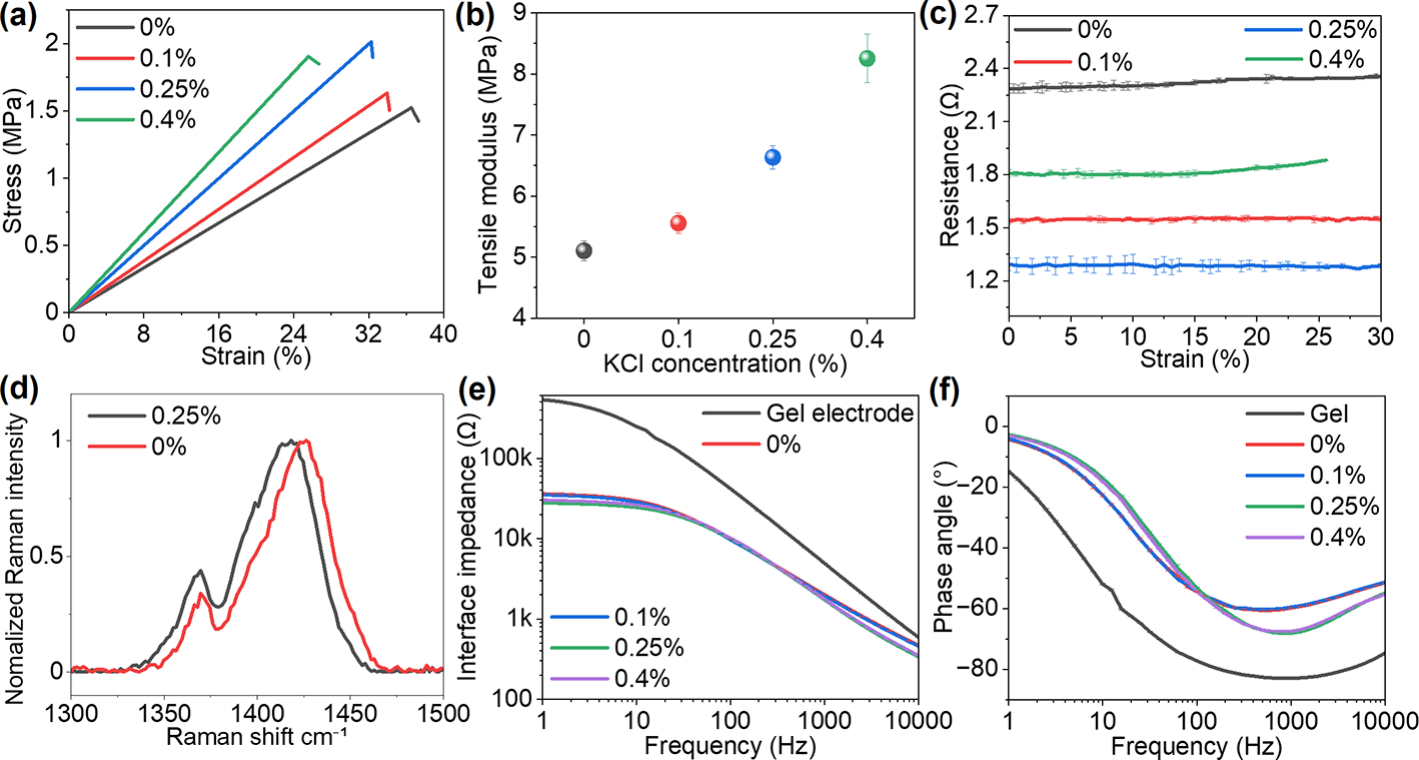
Nanocomposite mechanical properties and skin-electrode interface impedance. (a) Tensile stress–strain curves of freestanding nanocomposites with varying KCl concentrations and (b) derived nanocomposite tensile modulus. (c) Nanocomposite resistance changes with increasing strains (*n* = 3). (d) Raman spectra of the nanocomposite with and without KCl. (e) Comparison of the skin-electrode interface impedance measured with conventional Ag/AgCl gel electrodes and nanocomposite electrodes and corresponding (f) phase angles.

Low skin-electrode interface impedance is important for high-quality biosignal recording and efficient electrical stimulation.^2^ The skin-electrode interface can be modeled with an equivalent circuit comprising a series resistance (*R*_u_) and a parallel branch of a resistance (*R*_e_) with a constant-phase element (CPE) to account for interfacial heterogeneity (Figure S3a).^30^ In this model, *R*_u_ represents the resistance of the underlying tissue, while *R*_e_ corresponds to the charge-transfer resistance at the skin surface. The CPE captures the nonideal capacitive behavior of the interface. We measured the skin-electrode interface impedance as a function of frequency using electrochemical impedance spectroscopy (EIS) with nanocomposite electrodes and conventional Ag/AgCl gel electrodes (Figure 2e). In these measurements, the effective contact area was kept identical across all electrodes, ensuring that variations in interface impedance could be attributed solely to differences in electrode composition and morphology. All nanocomposite electrodes exhibit a much lower interface impedance in the frequency range from 1 Hz to 10 kHz, which is relevant to biosignal and stimulation frequency. The nanocomposite electrode with 0.25% KCl exhibited the lowest impedance compared to other composites over the entire frequency range. Compared to the gel electrode, the nanocomposite electrode with a 0.25% KCl exhibited an impedance 19.5 times lower at 1 Hz and 4.2 times lower at 100 Hz. While most wearable electrodes with optimized material and mechanical designs achieved interface impedance comparable to commercial Ag/AgCl gel electrodes,^31,32^ our electrodes showed much lower impedance. Such a low impedance with the nanocomposite electrodes mainly results from enhanced mixed ionic and electronic transport in PEDOT:PSS and a seamless interface. Adding a small amount of KCl reduced the interface impedance compared to that without KCl. Additionally, in the Bode phase response, the gel electrode exhibited a larger absolute phase angle than all nanocomposite electrodes across the measured frequency spectrum (Figure 2f). These results suggest that the nanocomposites support a more balanced charge-transfer mechanism at the skin-electrode interface, arising from the coexistence of ionic and electronic conduction pathways. The corresponding Nyquist plots for gel and nanocomposite electrodes are shown in Figure S3d–f. The parameters fitted with the equivalent circuit are summarized in Table S1. Nanocomposite electrodes exhibit lower charge-transfer resistance (*R*_e_) compared to gel electrodes, indicating more efficient charge transport at the skin-electrode interface. The constant phase element magnitude (*Y*_0_) for the nanocomposite electrodes is about an order of magnitude higher than that of the gel electrode, indicating its enhanced capacitive coupling. The reduced α values suggest a distributed capacitive behavior associated with volumetric ionic-electronic coupling within the conductive polymer network. In contrast, the series resistance (*R*_u_) remains comparable across electrodes, indicating consistent bulk tissue resistance and attributing the impedance differences primarily to the skin-electrode interface.

### Electrophysiological Recording with a Nanocomposite Interface

We recorded electrophysiological signals, including ECG and EMG, with nanocomposite and commercial Ag/AgCl gel electrodes for comparison. ECG measures the heart’s electrical activity and is routinely employed to diagnose disorders such as atrial fibrillation and myocardial infarction.^33^ EMG records the bioelectrical signals generated by skeletal muscles, providing insight into muscle performance and the neural drive underlying their contraction and relaxation. Beyond diagnostic use, EMG signals are widely applied as control inputs for human-machine interfaces.^34^ Nanocomposite and commercial gel electrodes were placed on the chest of human subjects to record ECG signals using a standard three-electrode configuration (Figure S4). Figure 3a,b and Figure S5a–c show ECG signals recorded with gel and nanocomposite electrodes with different KCl concentrations. Clear P, QRS, and T-wave features were observed across all electrodes. The gel electrode produced a QRS amplitude of 2.6 mV, while nanocomposite electrodes with 0.25% KCl provided a higher QRS amplitude of 2.8 mV. For nanocomposite electrodes with 0%, 0.1%, and 0.4% KCl, the amplitude decreased to 2.1–2.5 mV (Figure S5d). The signal-to-noise ratio (SNR) of these electrodes followed a similar trend with 41.6 dB for gel, 42.4 dB for 0% KCl, 42.3 dB for 0.1% KCl, 50.1 dB for 0.25% KCl, and 39.4 dB at 0.4% KCl (Figure 3c). These results suggest that the nanocomposite electrodes with 0.25% KCl yielded the highest SNR and QRS amplitude in ECG recordings. We examined the effect of electrode thickness on ECG signal quality using nanocomposite electrodes with thicknesses of ∼60, 120, and 300 μm. Comparable SNR values were observed across all thicknesses (Figure S6a,b). These results indicate that the low-modulus nanocomposite maintains intimate contact with the skin and provides a similar effective contact area independent of electrode thickness.

**Figure 3:**
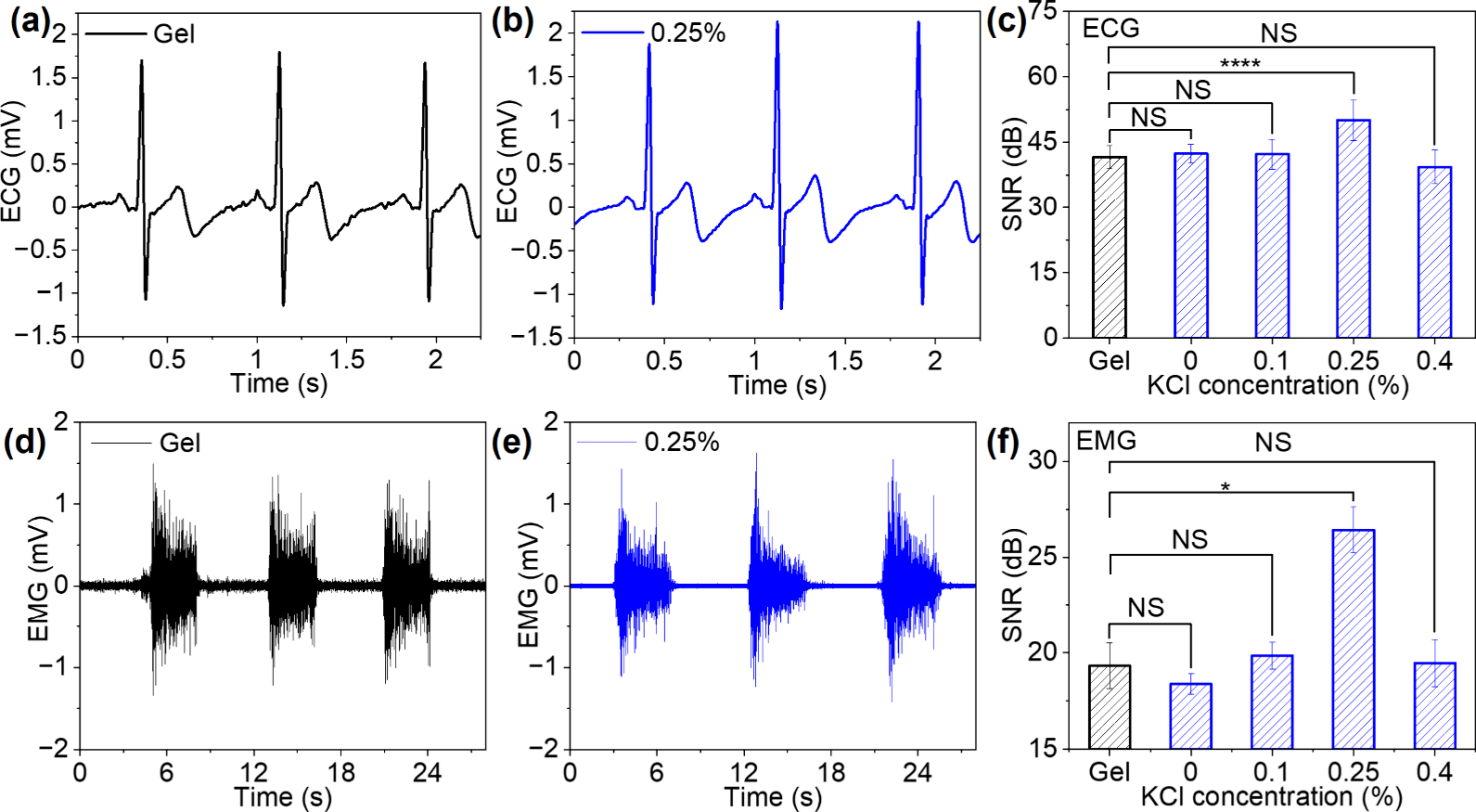
ECG and EMG recordings. (a, b) ECG signals measured with gel and nanocomposite electrodes. (c) SNR comparison between the gel and nanocomposite electrodes. *P*-values for SNR (gel vs 0%, gel vs 0.1%, and gel vs 0.4%), NS, and gel vs 0.25%, 3.99 × 10^–6^. (d, e) EMG signals measured with gel and nanocomposite electrodes. (f) SNR comparison between the gel and nanocomposite electrodes. *P*-values for SNR (gel vs 0%, gel vs 0.1%, and gel vs 0.4%), NS, and gel vs 0.25%, 0.02514. Data are presented as mean ± SD (*n* = 3). Statistical significance: *P < 0.05 and ***P < 0.0001, and NS indicates not significantly different.

To evaluate electrode performance under dynamic conditions, ECG signals were recorded while the subject performed a typing task involving continuous hand and upper-body motion. The nanocomposite electrode retained a higher SNR than the gel with no detectable motion artifacts (Figure S7a–c), demonstrating reliable signal acquisition under dynamic conditions. We also compared ECG signals immediately after nanocomposite electrode application and after 8 h of continuous wear (Figure S8a,b). The electrodes maintained clear ECG waveforms with well-defined features after prolonged use. The SNR did not exhibit a significant difference after 8 h (Figure S8c). After 8 h of continuous wear, no visible signs of skin irritation, redness, or inflammation attributable to the nanocomposite electrode were observed (Figure S9). These results indicate that the nanocomposite electrode maintains a stable skin-electrode interface and preserves signal fidelity during prolonged electrophysiological monitoring.

We performed EMG measurements by placing a pair of electrodes on the flexor muscles of the forearm to record muscle-contraction intensity induced by a fixed gripping force (Figure S10). EMG signals were continuously collected with nanocomposite and gel electrodes during three cycles of 3 s muscle contraction followed by 5 s rest (Figure 3d,e and Figure S11a–c). We calculated the signal and noise of EMG recordings from the rectified voltages during muscle contraction and rest using root-mean-squared (RMS) analysis (Figure 3f). The gel electrode exhibits a high background noise of 30 μV and an SNR of 19 dB. In contrast, nanocomposite electrodes provide higher-quality recordings, with noise levels decreased to 24 μV at 0% and 0.1% KCl, 12 μV at 0.25% KCl, and 25 μV at 0.4% KCl (Figure S11d). The nanocomposite electrode with 0.25% KCl shows 2.5 times lower noise than the gel electrode. The SNR of nanocomposite electrodes increased to 19–20 dB at 0.1% KCl and 0.4% KCl, and a maximum of 26 dB at 0.25% KCl. The 0.25% KCl formulation shows the EMG bursts with the highest SNR and the lowest noise among all the electrodes. We further evaluated the stability of EMG recordings during prolonged electrode wear for up to 10 h (Figure S12). For the gel electrode, the noise gradually increased to 38 μV after 10 h, accompanied by a decrease in SNR to 12 dB. In contrast, the 0.25% KCl nanocomposite electrode exhibited lower noise (∼8 μV) and higher SNR (∼29 dB), which stabilized after 6 h. These results demonstrate that the nanocomposite electrodes provide higher signal quality and better long-term stability than gel electrodes.

### Bioimpedance-Based Hemodynamic Recording Using a Nanocomposite Interface

Bioimpedance measurements can be employed to noninvasively monitor hemodynamic parameters, such as blood pressure and pulse wave velocity, providing real-time insight into cardiovascular function.^22,35^ Continuous, noninvasive monitoring of cardiovascular dynamics requires electrodes that enable a conformal skin interface, low interface impedance, and long-term stability to detect subtle hemodynamic features accurately.^36^ Rigid metal electrodes often fail to meet these requirements, exhibiting high electrode-skin impedance and noisy pulse waveforms, while widely used gel electrodes have limitations in long-term stability and signal fidelity. We evaluated the performance of nanocomposite electrodes with varying KCl concentrations for bioimpedance recording in comparison with commercial gel electrodes. Four electrodes of each type were applied along the radial artery at the wrist to enable continuous bioimpedance recording (Figure 4a). The two outer electrodes delivered a 400 μA alternating current at 100 kHz, while the two inner electrodes recorded the resulting voltage response. The measured voltage was subsequently converted into impedance values as a function of time. Bioimpedance waveforms over four pulse periods obtained using both gel and nanocomposite electrodes are shown in Figure 4b,d, and Figure S13a–c. The baseline impedance primarily reflects the bulk resistivity of the underlying tissue layers, dominated by skin and subcutaneous composition, while dynamic fluctuations superimposed on this baseline correspond to pulsatile hemodynamic changes. The baseline impedance measured with these electrodes fluctuated between 45 Ω and 75 Ω, with the lowest at 0.25% KCl nanocomposite electrodes (∼48 Ω) and the highest at conventional gel electrodes (∼70 Ω). The difference likely results from the effect of interface impedance. The first derivative of the impedance magnitude (d*Z*/d*t*) was calculated and plotted with positive polarity (Figure 4c,e, and Figure S13d–f). The d*Z*/d*t* signal can be used to extract fundamental hemodynamic parameters such as stroke volume and cardiac output.^37^ In the bioimpedance pulse waveform recorded with the nanocomposite electrode, three characteristic peaks are clearly distinguishable: the percussion (P) wave, the tidal (T) wave, and the dicrotic (D) wave (Figure 4d). These peaks correspond to blood ejection from the left ventricle into the aorta, reflected waves from the upper and lower body, and closure of the aortic valve, respectively.^38^ In contrast, recordings obtained with gel electrodes produced bioimpedance waveforms in which the T and D waves were less clearly distinguishable. The radial augmentation index (AIr), defined as the ratio of the P wave amplitude to the T wave amplitude, is a widely used parameter for assessing arterial stiffness.^38^ We calculated the AIr to be ∼40% from the bioimpedance waveform obtained with the nanocomposite electrodes, which is consistent with the reported range of 40–60% for healthy young adults.^39,40^ We compared the maximum d*Z*/d*t* amplitudes derived from bioimpedance waveforms recorded with nanocomposite and gel electrodes (Figure 4f). The maximum d*Z*/d*t* amplitudes reached the highest of 3.22 Ω/s for the 0.25% KCl nanocomposite electrodes, which was approximately 3.7 times higher than that of gel electrodes. These results confirm that the nanocomposite electrodes with an optimal ionic loading provide the highest sensitivity in capturing rapid hemodynamic changes.

**Figure 4:**
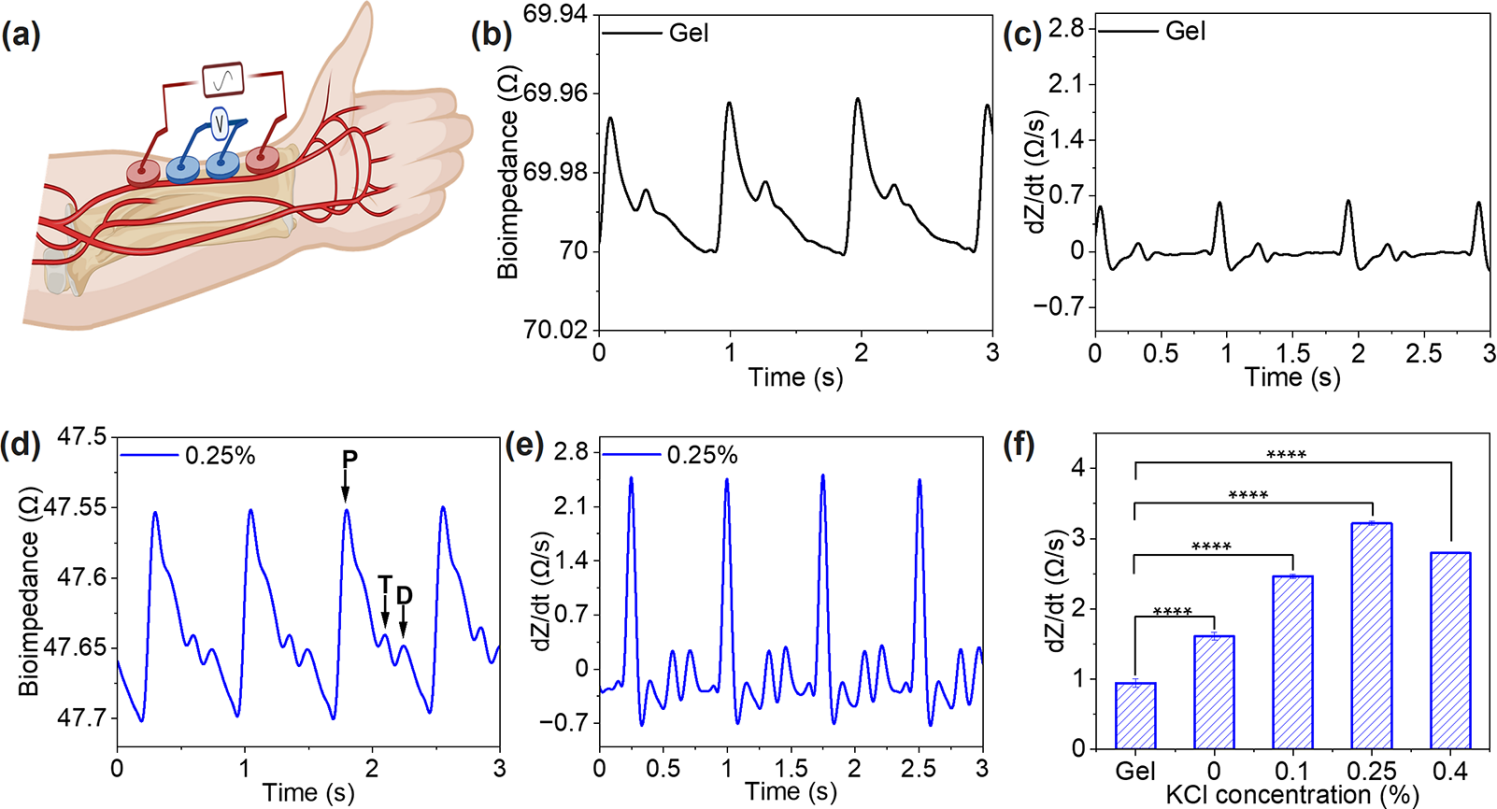
Bioimpedance recordings. (a) Schematic illustration showing bioimpedance recordings along the radial artery at the wrist. (b) Bioimpedance signals and (c) the first derivative with respect to time measured Ag/AgCl gel electrodes. (d) Bioimpedance signals and (e) the first derivative with respect to time measured with nanocomposite electrodes. (f) Comparison of the peak height of the d*Z*/d*t* of the gel and the nanocomposite electrode. *P*-values for gel vs 0% (3.48 × 10^–6^), gel vs 0.1% (2.42 × 10^–7^), gel vs 0.25% (6.14 × 10^–8^), and gel vs 0.4% (3.69 × 10^–6^). Data are presented as mean ± SD (*n* = 3). Statistical significance: ****P < 0.0001.

### Transcutaneous Electrical Stimulation with Nanocomposite Interfaces

Transcutaneous electrical stimulation is widely used in neuromodulation, rehabilitation, and pain management due to its ability to noninvasively modulate neural activity through the skin.^41,42^ Conventional hydrogel electrodes are commonly used in these clinical applications, but have limited charge injection capacity and stimulation comfort.^43^ Compared with conventional electrodes, our electrodes can enhance charge-injection capacity and extend the tolerance threshold, owing to the nanocomposite’s combined ionic and electronic conductivity. We first assessed charge injection capacity (CIC) ex vivo using pig skin. We recorded voltage transients to quantify the CIC by injecting charge-balanced, cathodal-first, symmetric biphasic current pulses with 200 μs phase duration (Figure 5a). This protocol is widely used in neural stimulation studies to minimize irreversible Faradaic reactions and tissue damage.^27^ The current limit for reversible charge injection was defined by the water hydrolysis window (−0.9 to 0.6 V for PEDOT:PSS), with cathodal and anodal potentials determined 10 μs after pulse termination (Figure 5b). Voltage transients and current responses of charge-balanced biphasic pulses were collected with commercial stimulation gel electrodes (Axelgaard PALS) and nanocomposite electrodes for comparison. The 0.25% KCl nanocomposite electrodes achieved the CIC of 1.33 μC/cm^2^, corresponding to a 2.6-fold increase over gel electrodes (0.51 μC/cm^2^). The 0% and 0.1% KCl nanocomposites also enhanced CIC to 1.03–1.19 μC/cm^2^ by ∼2-fold, whereas excess ionic loading at 0.4% reduced CIC to 0.82 μC/cm^2^ (1.6-fold) (Figure 5c). Collectively, these results confirm that the 0.25% KCl nanocomposite electrode provides the highest electrical stimulation efficiency in comparison with other nanocomposite and gel electrodes.

**Figure 5:**
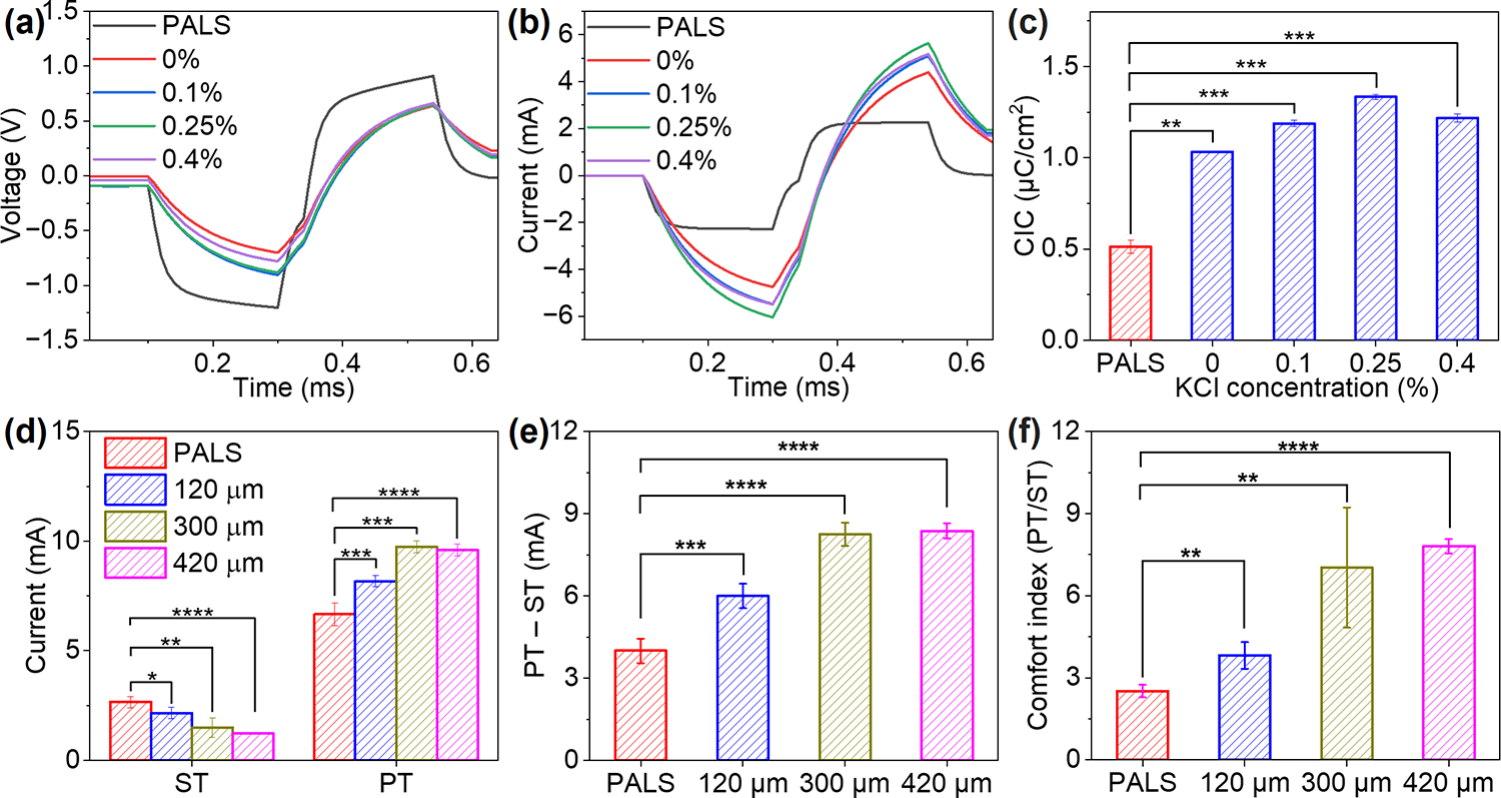
Electrochemical and transcutaneous stimulation performance of PALS and nanocomposite electrodes. (a, b) Representative voltage transient curves and corresponding current pulses recorded from PALS and nanocomposite electrodes with varying KCl concentrations (0, 0.1, 0.25, and 0.4%). (c) Calculated charge injection capacities (CIC) for PALS and nanocomposite electrodes, showing enhanced charge delivery with nanocomposites.*P*-values (PALS vs nanocomposite): 0.00256 (0% KCl), 3.25× 10^–4^ (0.1% KCl), 2.89× 10^–4^ (0.25% KCl), and 2.44× 10^–4^ (0.4%KCl). (d) Sensation threshold (ST) and pain threshold (PT) currents for PALS gel and nanocomposite electrodes with different thicknesses. *P*-values (PALS vs nanocomposite): ST = 0.04086 (120 μm), 0.00863 (300 μm), and 3.29 × 10^–5^ (420 μm); PT = 4.35 × 10^–4^ (120 μm), 1.51 × 10^–4^ (300 μm), and 2.97 × 10^–6^ (420 μm). (e) Stimulation window (PT-ST) for PALS gel and nanocomposite electrodes. *P*-values (PALS vs nanocomposite): 1.10 × 10^–4^ (120 μm), 1.29 × 10^–5^ (300 μm), and 2.18 × 10^–8^ (420 μm). (f) Comfort index (PT/ST) for PALS gel and nanocomposite electrodes. *P*-values (PALS vs nanocomposite): 0.00148 (120 μm), 0.00476 (300 μm), and 9.59 × 10^–10^ (420 μm). All error bars denote SD, and NS represents not significant. Statistical significance: *P < 0.05, **P < 0.01, ***P < 0.001, and ****P < 0.0001.

We next evaluated the effectiveness of transcutaneous electrical stimulation in human subjects. Stimulation was delivered as charge-balanced, cathodal-first biphasic current pulses (200 μs per phase) using surface electrodes placed on the ventral side of the left forearm. Sensation threshold (ST) was defined as the lowest current intensity at which a participant reported perceiving any sensation, whereas pain threshold (PT) was defined as the visual analog scale (VAS) ≥ 3/10, a threshold commonly used in experimental pain research to define more clearly perceptible discomfort.^44^ In these experiments, participants were blinded to the type of stimulation intensity. Both commercial gel and nanocomposite electrodes reliably elicited ST and PT (Figure 5d). We prepared 0.25 wt % KCl nanocomposite electrodes with thicknesses ranging from 120 to 420 μm to evaluate stimulation efficiency and comfort. Both commercial gel and nanocomposite electrodes reliably elicited ST and PT. The PALS gel electrode exhibited a stimulation window (PT-ST) of 4 mA (Figure 5e). In comparison, the nanocomposite electrodes expanded the stimulation window to 8 mA, representing a 100% increase in operating range (Figure 5e). The comfort index (PT/ST) increased from 2.5 for gel electrodes to 7 for nanocomposite electrodes, corresponding to a 2.8-fold improvement in tolerance (Figure 5f). These results suggest that thicker nanocomposite electrodes enable more efficient charge injection and significantly improve stimulation comfort. Stimulation comfort strongly depends on electrode-skin interfacial properties, including impedance, polarization behavior, and current distribution at the skin surface.^45^ Under current-controlled stimulation, these properties reduce polarization voltage and promote volumetric ionic-electronic charge coupling, providing the expanded stimulation window and higher pain thresholds observed here.

To evaluate robustness during repeated use, we performed 30 reapplication cycles, where in each cycle the electrode was reapplied to the skin, the stimulation protocol was conducted, and the electrode was subsequently removed. We then compared the stimulation performance between early cycles (1–3) and late cycles (28–30) (Figure S14a–c). The stimulation window and comfort index also showed no significant differences between early and late cycles. These results demonstrate that the nanocomposite electrode maintains reproducible performance during repeated stimulation and use. Stability testing further demonstrated that these advantages were retained after 7 days of ambient storage (Figure S15a–c). The reduced stimulation efficiency of gel electrodes is attributed to drying-induced water loss and impaired ionic transport, which increase interfacial impedance and narrow the safe operating range. In contrast, hygroscopic dopants in the nanocomposite electrodes preserve hydration and efficient ionic-electronic transport, providing enhanced stimulation stability. These findings underscore the robustness and durability of nanocomposite electrodes compared to commercial gel electrodes, establishing them as an efficient and comfortable interface for long-term wearable neuromodulation. Although nanocomposite electrodes demonstrated improved recording quality and stimulation efficiency relative to commercial gel electrodes, the evaluation was conducted under a limited set of stimulation and wear conditions. Long-term performance under extended continuous wear, repeated sweating, motion, and environmental exposure remains to be established. Potential improvements include enhanced encapsulation and adhesion to support long-term wear, as well as integration of the electrodes into fully wearable systems with standardized readout and stimulation modules.

## Conclusions

We developed a soft, stretchable PEDOT:PSS-based nanocomposite incorporating multifunctional additives to realize a stable, low-impedance, and efficient skin-electrode interface for electrophysiological recording and transcutaneous stimulation. The synergistic combination of hygroscopic and ionic dopants enables balanced ionic-electronic transport, mechanical softness, and long-term hydration stability. The optimized nanocomposite electrodes deliver higher signal-to-noise ratios in ECG and EMG recordings, enhanced bioimpedance sensitivity, and an expanded safe and comfortable stimulation window. These findings establish a materials design framework for soft, durable, and high-fidelity bioelectronic interfaces, advancing next-generation wearable sensing and neuromodulation technologies.

## Experimental Section

### Materials

PEDOT:PSS aqueous solution (Clevios PH1000) was purchased from Heraeus Epurio LLC. PDMS (Sylgard184) was obtained from Dow Corning. d-Sorbitol, glycerol, and potassium chloride were purchased from Thermo Scientific Chemicals. Aqueous solutions were prepared with DI water (18.2 MΩ-cm) produced by the Sartorius Arium Pro Ultrapure water system. All chemicals were used as purchased.

### Fabrication of Skin-like Nanocomposite Electrodes

d-Sorbitol (3% w/v) and glycerol (3.5% w/v) were mixed with a pristine PEDOT:PSS aqueous solution (1.1–1.3 wt %) to obtain a plasticized PEDOT:PSS solution. The mixtures were vigorously vortexed for 2 min, sonicated for 20 min, and then shaken for an additional 20 min to ensure homogeneity. KCl was then incorporated into the plasticized PEDOT:PSS solution at concentrations of 0, 0.1, 0.25, and 0.4% (w/v), followed by an additional 30 min of shaking to achieve uniform ionic incorporation. To prepare freestanding electrodes, a PDMS prepolymer and curing agent (in a 10:1 ratio) were spin-coated onto polyimide substrates at 500 rpm for 1 min and then cured at 125 °C for 20 min. The cured PDMS substrates were plasma-treated to enhance surface wettability. The Parafilm was patterned with openings using a CO_2_ laser cutter and then laminated onto the PDMS surface to confine the PEDOT:PSS solution within designated regions. The nanocomposite precursors were drop-cast into designated regions and allowed to dry at room temperature. After removing the Parafilm, the patterned nanocomposite films were subsequently dried at 60 °C and annealed at 130 °C, yielding soft, freestanding electrodes with precisely defined geometries. Tegaderm served as a stretchable backing layer for the electrodes with the interconnect area encapsulated for mechanical stability and electrical insulation. The electrodes were connected to the data acquisition system using flexible, low-voltage, high-temperature silicone-insulated wires (36 AWG, McMaster-Carr) for all experiments.

### Nanocomposite Film Characterization

The tensile stretch measurement was conducted using MARK-10. The applied strain rate during the tensile testing was 3 mm/min. The thin film resistance was measured using a digital multimeter (NI-USB4065) with a four-probe method. The voltage transient response and skin-electrode impedance were measured using a Gamry Reference 600+ potentiostat, with tests performed on pig skin and human skin, respectively. A three-electrode system was used. All electrodes utilized for electrochemical characterization have a geometric surface area (GSA) of 1.767 cm^2^. The pulse duration for the voltage transient measurement was 200 μs. We analyzed the CIC by applying the biphasic symmetric, cathodal-first current pulses over the working and counter electrodes. The maximum cathodal and anodal electrochemical potentials were determined as the potentials at 10 μs after the cathodal and anodal pulses ended, following the previously reported protocol.^27^ CIC was calculated using eq 1, where total delivered (injected) charge, *Q*_inj_ (mC) = *Q*_inj(cathodal phase)_ + *Q*_inj(anodal phase)_

$$\mathrm{CIC}=\left(\frac{Q_{\mathrm{inj}}}{\mathrm{GSA}}\right)$$ 1

Impedance was analyzed using EIS with a frequency range of 1 Hz to 10 kHz and a potential bias of 10 mV.

### Electrophysiological Measurement

Electrocardiography (ECG) and electromyography (EMG) were recorded using the BIOPAC system (MP160, ECG 100D, and EMG 100D). For ECG measurements, three electrodes (positive, negative, and ground) were placed on the chest in a Lead II-type configuration, with RA (VIN−) near the right clavicle, LL (VIN+) on the left lower rib region, and RL (GND) on the right lower rib region. Signals were sampled at 2 kHz, notch-filtered at 60 Hz to suppress powerline interference, and band-pass filtered between 0.5 and 35 Hz. Bipolar EMG signals were collected from the forearm muscles using two electrodes placed along the ventral forearm and a ground electrode near the wrist. Raw EMG signals (10 kHz sampling rate) were notch-filtered at 60 Hz to suppress powerline interference, band-pass filtered between 20 and 450 Hz, and smoothed using a 25 ms RMS moving window to quantify muscle activation. The SNR was calculated using eq 2. For ECG analysis, *A*_signal_ represents the RMS amplitude of the QRS complex, and *A*_noise_ denotes the RMS value of the non-QRS region. A template-subtraction method was used, in which aligned beats were averaged to form a representative waveform, and noise was defined as the residual after subtraction of individual beats, thereby isolating nonreproducible components such as electrode noise and motion artifacts that vary across cycles. For EMG analysis, *A*_signal_ corresponds to the RMS amplitude during muscle contraction, while *A*_noise_ represents the RMS value during muscle rest.

$$\mathrm{SNR}\left(dB\right)=20\log_{10}\left(\frac{A_{\mathrm{signal}}}{A_{\mathrm{noise}}}\right)$$ 2

### Hemodynamic Measurement

Bioimpedance was assessed using a BIOPAC system (MP160, NICO100C) in a tetrapolar configuration. Four electrodes were positioned longitudinally along the radial artery near the wrist with a 1 cm center-to-center spacing. The effective contact area was identical for the commercial gel and nanocomposite electrodes. Electrodes were applied directly to clean, dry skin without abrasive or chemical skin preparation. Participants were seated with their forearms resting at heart level and instructed to remain still during data acquisition to minimize hydrostatic pressure variations. A 400 μA current was applied through the outer electrodes, while the inner electrodes measured the voltage response, which was subsequently converted into impedance. The signals were filtered using a 10 Hz low-pass filter and a DC high-pass filter. The study involving human subjects received approval from the Texas A&M University Institutional Review Board (IRB2019-0811F). All participants provided written informed consent prior to enrollment, including consent for the use of photographs, following a thorough explanation of the study procedures and potential risks.

### Transcutaneous Electrical Stimulation

Electrical stimuli were delivered using a constant-voltage STIMISOLA isolated stimulator (BIOPAC Systems Inc., Goleta, CA, USA) through commercial PALS gel electrodes (Axelgaard Manufacturing, USA) and nanocomposite electrodes (0.25% KCl), both with identical sizes (area = 1.767 cm^2^). The proximal electrode was placed 0.5 in. below the elbow on the ventral side of the left forearm, and the distal electrode was positioned on the thenar eminence of the left hand, lateral to the midline and distal to the wrist. Electrodes were connected to the stimulator with the positive terminal proximally and the negative terminal distally. Charge-balanced biphasic cathodic-first pulses (0.4 ms pulse width) were applied at 100 Hz in trains of five pulses, repeated in five trains with refractory intervals. Sensory threshold (ST) was defined as the first consistent paresthesia, and pain threshold (PT) as a rating of ≥ 3 on a 10-point visual analog scale.^46^ The stimulation window (PT-ST) and comfort index (PT/ST) were calculated for comparison.

## Supplementary Material



## Data Availability

All data needed to evaluate the conclusions in the paper are present in the paper and the Supporting Information.

## Notes

Figure S1: Nanocomposite with selective omission of d-sorbitol or glycerol at 0.25% KCl concentration; Figure S2: Cross-sectional morphology of nanocomposite films with different compositions; Figure S3: Equivalent circuit model and impedance characterization of electrode-skin interfaces. Table S1: Summary of fitted parameters using the equivalent circuit model; Figure S4: Photographs showing electrode placement for ECG acquisition using a Lead II-type configuration; Figure S5: ECG recordings with nanocomposite electrodes of varying KCl concentrations; Figure S6: Thickness-dependent ECG recording with nanocomposite electrodes of varying thicknesses (0.25% KCl); Figure S7: ECG recording during a dynamic activity (typing); Figure S8: ECG signal stability after 8 h of continuous wear; Figure S9: Photographs of the skin after 8 h of continuous electrode wear, comparing the nanocomposite and commercial gel electrodes; Figure S10: Representative photograph showing EMG electrode placement on the forearm; Figure S11: EMG recordings with nanocomposite electrodes of varying KCl concentrations; Figure S12: Stability of EMG recordings during prolonged wear; Figure S13: Bioimpedance measurements with nanocomposite electrodes of varying KCl concentrations; Figure S14: Electrical stimulation performance of nanocomposite electrodes (0.25% KCl) across repeated reapplication cycles; Figure S15: Stability of transcutaneous stimulation with reused PALS gel and nanocomposite electrodes (PDF)
