# Supplementary material for: Soft, Skin-Conformal Electronic Interfaces for Multimodal Biosignal Monitoring and Transcutaneous Stimulation
Source: ACS Appl Mater Interfaces. 2026 Apr 28;18(18):25863–72. doi: 10.1021/acsami.6c02518 (PMC13181717; doi:10.1021/acsami.6c02518)
Supplement: Supplementary file 1 [file am6c02518_si_001.pdf]

Supporting Information

Soft, Skin-Conformal Electronic Interfaces for Multimodal Biosignal Monitoring and Transcutaneous Stimulation

Md Saifur Rahman[1], Ziyu Zhu[1], Nicholas B. Abadie[1], Kailash Pillai[2], Adam Reyes[1], Luke McKinley[1], Creed G. Bielss[1], Salina Teng[2], Jie Zhao[1], Dimitry G. Sayenko[3], Limei Tian[1*]

[1]Department of Biomedical Engineering, Center for Remote Health Technologies and Systems, Texas A&M University, College Station, TX 77843, USA.

[2]Department of Electrical and Computer Engineering, Texas A&M University, College Station, TX 77843, USA.

[3]Department of Neurosurgery, Center for Neuroregeneration, Houston Methodist Research Institute, Houston, TX 77030, USA.

*Corresponding author. Email: ltian@tamu.edu

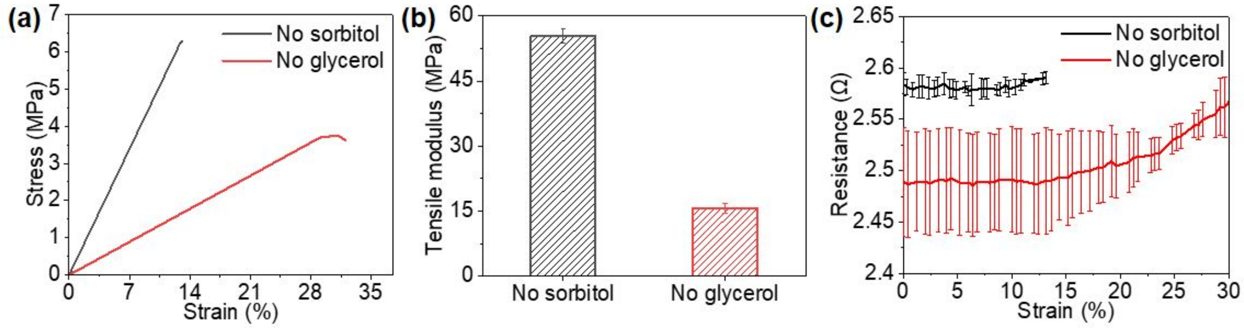

Figure S1. Nanocomposite with selective omission of D-sorbitol or glycerol at 0.25 wt%KCl concentration. (a) Tensile stress-strain curves of freestanding nanocomposite films without D-sorbitol and without glycerol, respectively and **(b)** derived nanocomposite tensile modulus. **(c)** Sheet resistance as a function of tensile strain (n = 3).

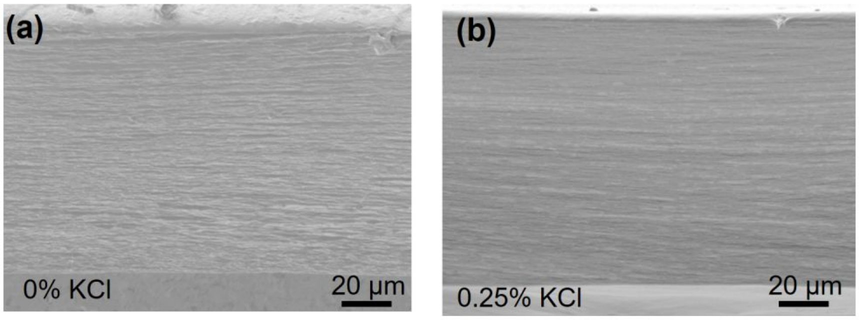

Figure S2. Cross-sectional morphology of nanocomposite films with different compositions. **(a)** PEDOT:PSS/sorbitol/glycerol. **(b)** PEDOT:PSS/sorbitol/glycerol/0.25% KCl.

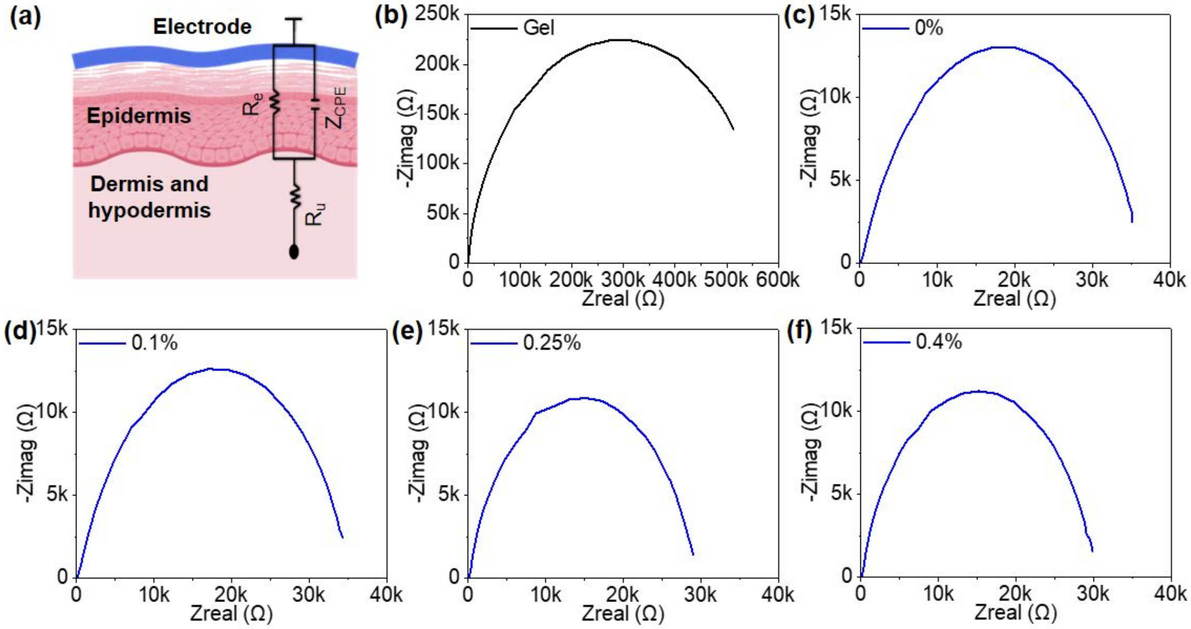

Figure S3. (a) Equivalent circuit model of the electrode-skin-body interface. **(b -f)** Nyquist plot of a commercial gel and nanocomposite electrodes.

Electrode area (1.767 cm^2)	R_e	R_u	Y_0	alpha
Gel	5.39E+05	8.01E+01	6.97E-08	9.14E-01
0%	4.01E+04	9.11E+01	8.61E-07	7.19E-01
0.1%	3.93E+04	8.91E+01	8.61E-07	7.23E-01
0.25%	1.83E+04	73.74	1.30E-06	6.66E-01
0.4%	2.93E+04	53.81	1.07E-06	6.65E-01

Table S1. Summary of fitted parameters using the equivalent circuit model. $\boldsymbol{R_e}$ represents the charge-transfer resistance between the surface of the skin and the electrode, $\boldsymbol{R_u}$ represents the resistance of the underlying tissue, and the impedance of the contact phase element CPE is Z_{CPE}=1 ((j) Y_0). Specifically, Y_0 is the magnitude of 1/Z_{CPE} at ω = 1

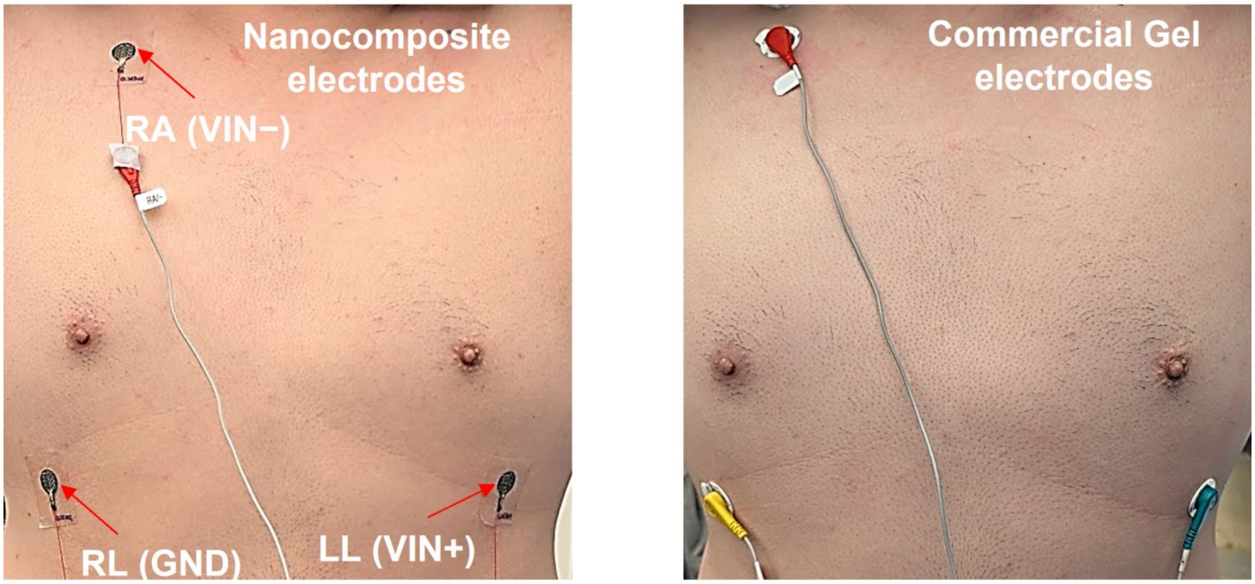

Figure S4. Photographs showing electrode placement for ECG acquisition using a Lead II-type configuration. The negative electrode **RA (VIN-)** was placed near the right clavicle, the positive electrode **LL (VIN+)** on the left lower rib region, and the reference electrode **RL (GND)** on the right lower rib region. Left: nanocomposite electrode. Right: commercial gel electrode used as the control.

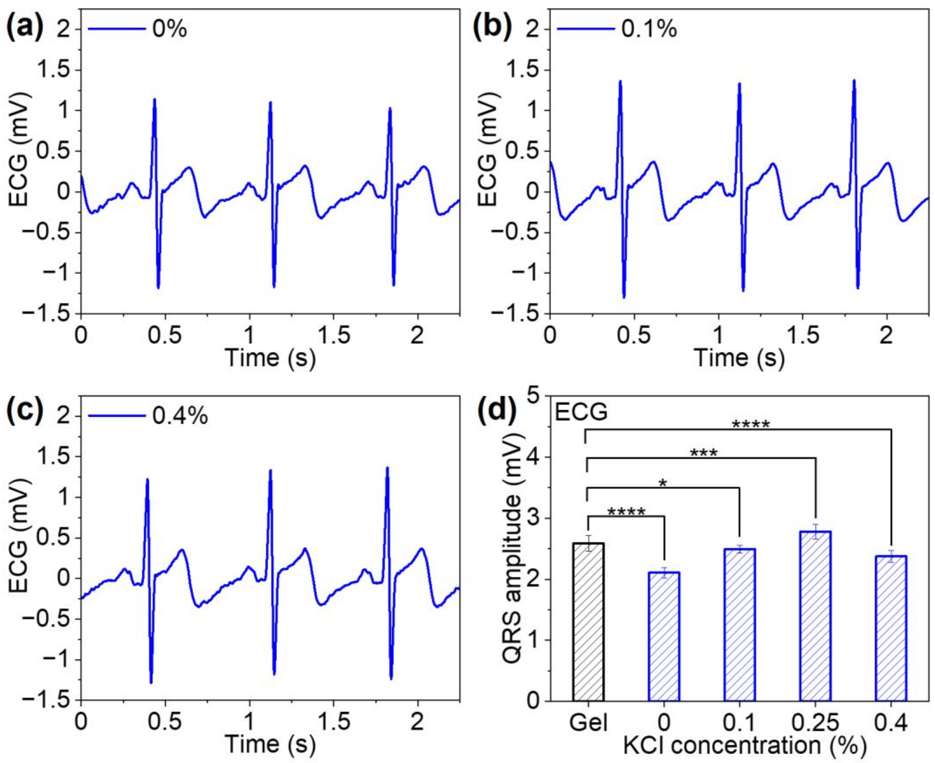

Figure S5. ECG recordings. (a-c) Representative ECG signals recorded using nanocomposite electrodes with different KCl concentrations. **(d)** Comparison of the RMS noise amplitude measured from the PR segment. **(e)** Comparison of QRS amplitude across gel and nanocomposite electrodes. Nanocomposite electrodes show improved signal quality compared to gel electrodes. Data are presented as mean ± SD (n = 3). Statistical significance: $^{*}P < 0.05$, $^{**}P < 0.01$, $^{***}P < 0.001$, and $^{****}P < 0.0001$; and NS indicates not significantly different.

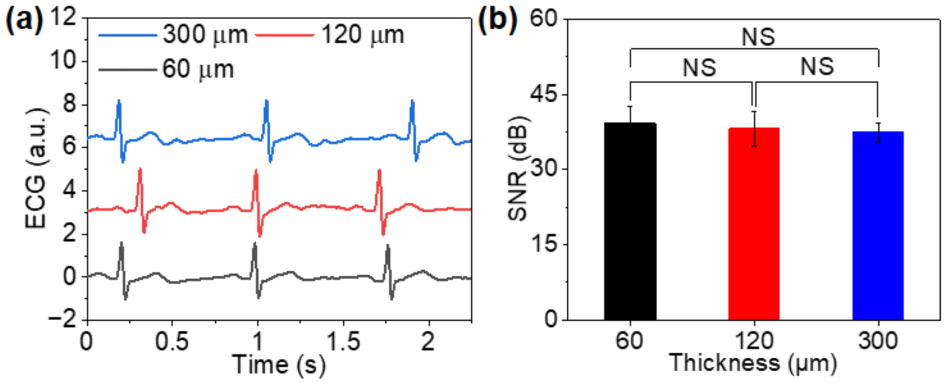

Figure S6. Thickness-dependent passive ECG sensing performance of nanocomposite electrodes (0.25% KCl). (a) Representative ECG recordings obtained using nanocomposite electrodes with thicknesses of ~60, ~120, and ~300 µm. **(b)** SNR of ECG recordings for the three thickness groups. All error bars denote the SD, and NS indicates not significantly different ($P \geq 0.05$). n = 3.

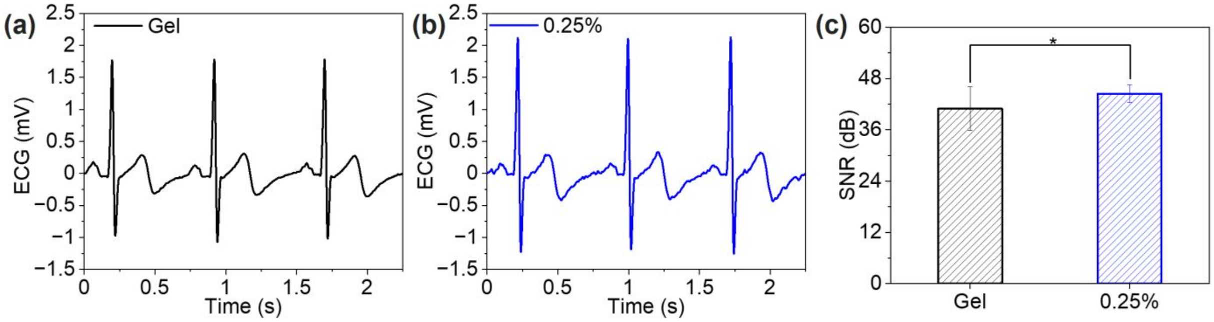

Figure S7. ECG recording during a dynamic activity (typing). (a) ECG signals recorded with a commercial gel electrode. **(b)** ECG signals recorded with the nanocomposite electrode (0.25% KCl). **(c)** SNR comparison during typing. Data are presented as mean ± SD (n = 3). Statistical significance: $P < 0.05$.

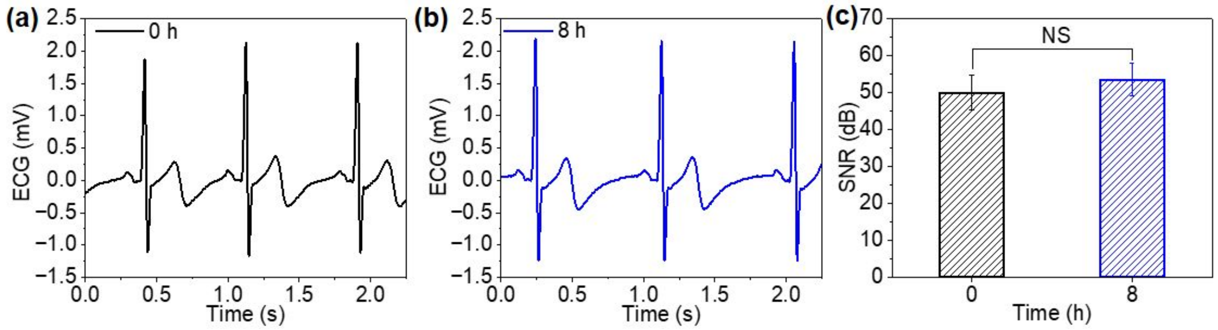

Figure S8. ECG signal stability after 8 h of continuous wear. (a,b) Representative ECG signals recorded immediately after application (0 h) and after 8 h using nanocomposite electrodes (0.25% KCl). **(c)** Signal-to-noise ratio (SNR) comparison. Data are presented as mean ± standard deviation (SD) (n = 3). NS, not significant.

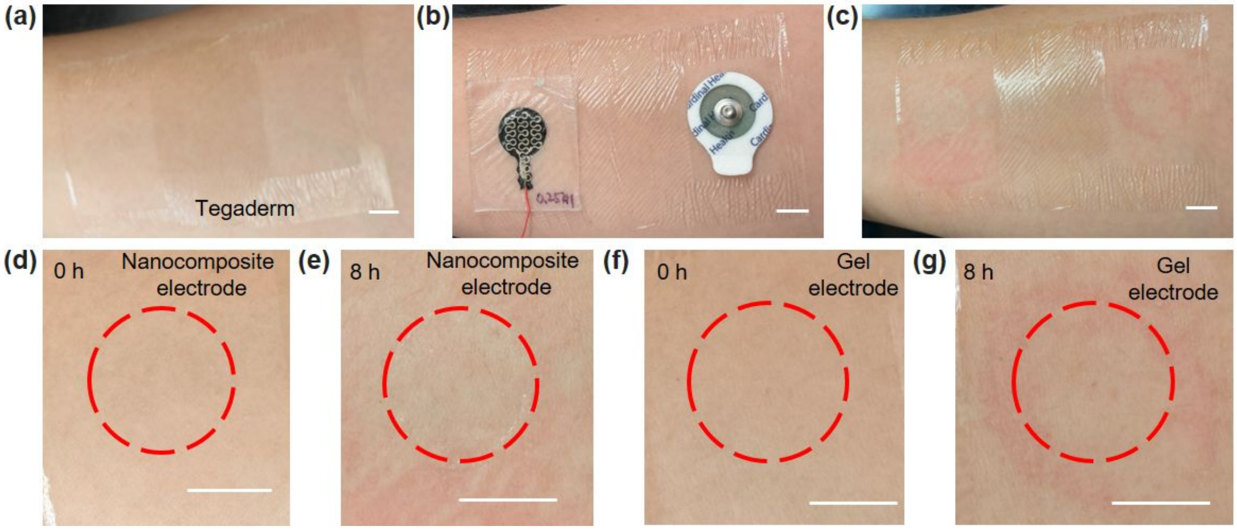

Figure S9. Photographs of the skin after 8 h of continuous electrode wear, comparing the nanocomposite and commercial gel electrodes. (a) Baseline skin condition prior to electrode application; the region of interest (ROI) was defined and enclosed using Tegaderm film. **(b)** Nanocomposite and commercial gel electrodes. **(c)** Skin appearance immediately after 8 h of wear and electrode removal. **(d,e)** Magnified views of the ROI before and after application of the nanocomposite electrode. **(f,g)** Magnified views of the ROI before and after application of the gel electrode. Dashed circles indicate the electrode contact regions. No visible skin irritation, erythema, or inflammation was observed. Minor redness surrounding the nanocomposite electrode is attributed to the adhesive, indicating good skin compatibility. In contrast, slight redness was observed beneath the gel electrode. Scale bars: 1 cm.

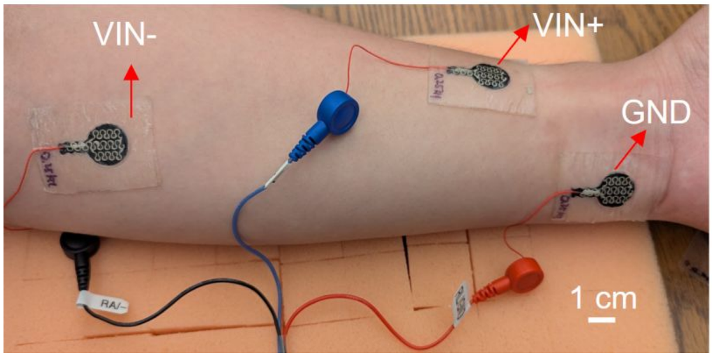

Figure S10. Representative photograph showing EMG electrode placement on the forearm. Two electrodes were positioned along the forearm muscle for differential recording (VIN+ and VIN-), while a ground electrode (GND) was placed near the wrist.

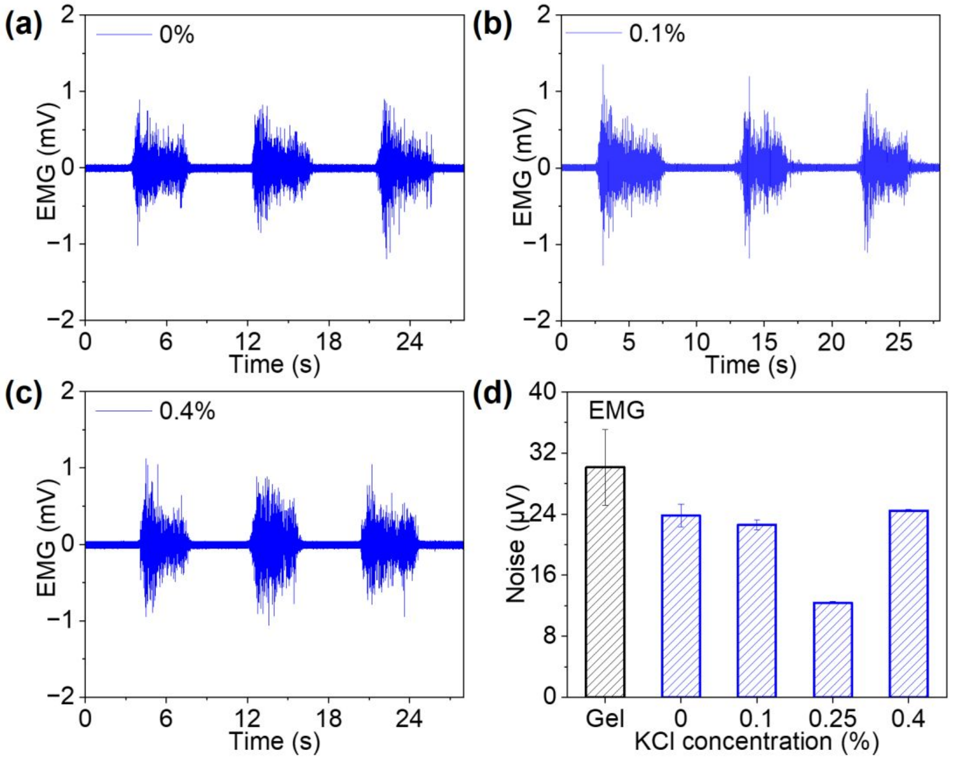

Figure S11: EMG recordings. (a-c) EMG signals were measured with nanocomposites. **(d)** Comparison of EMG noise levels for gel and nanocomposite electrodes, quantified as the RMS noise (μV) calculated from non-signal (baseline) segments.

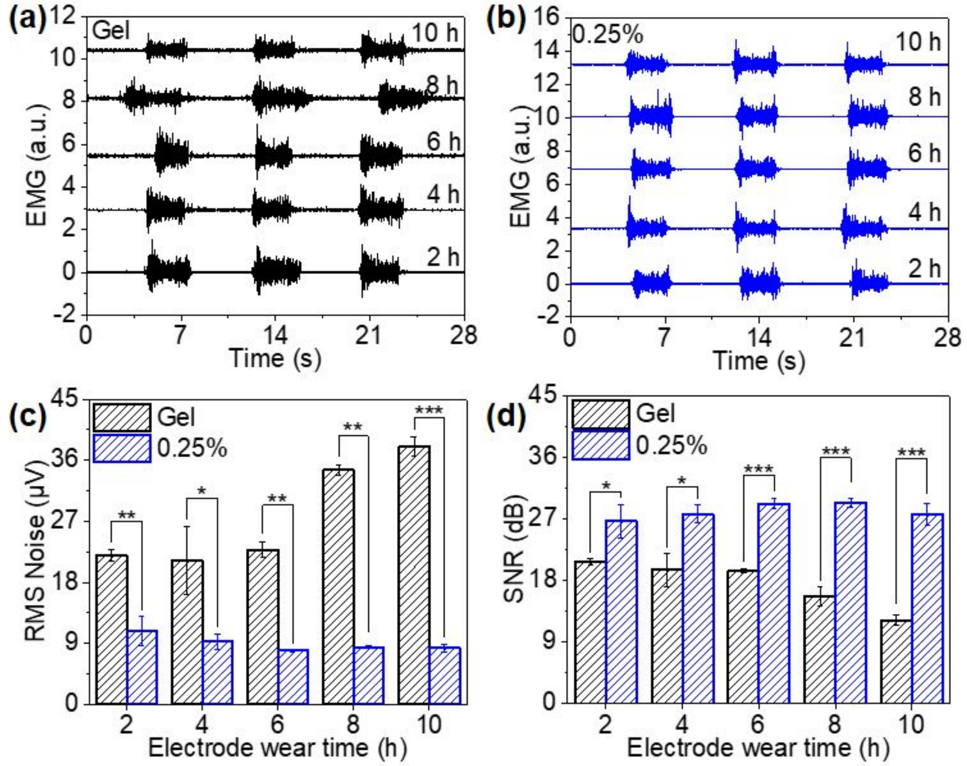

Figure S12. Stability of EMG recordings during prolonged wear. (a) Representative EMG signals recorded using commercial gel electrodes at 2, 4, 6, 8, and 10 h. **(b)** Representative EMG signals from nanocomposite electrodes (0.25% KCl) at the same time points. **(c)** Noise **(d)** SNR as a function of electrode wear time. Data are presented as mean ± SD (n = 3). Statistical significance: $^{*}P < 0.05$, $^{**}P < 0.01$, $^{***}P < 0.001$.

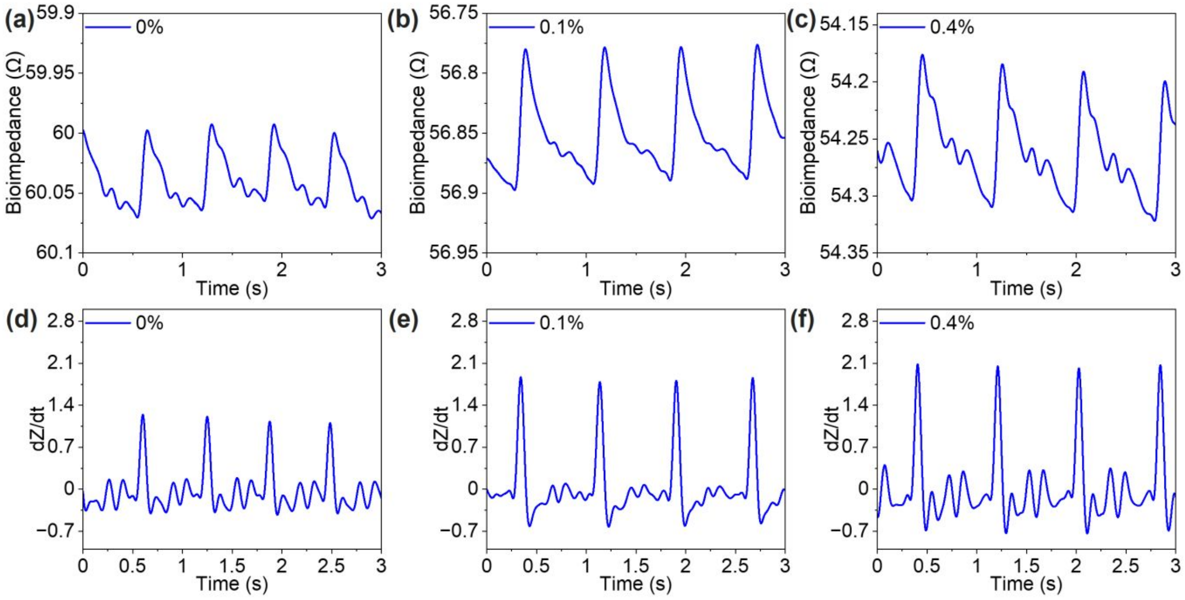

Figure S13. (a-c) Bioimpedance signals and **(d-f)** the first derivative with respect to time measured with nanocomposite electrodes.

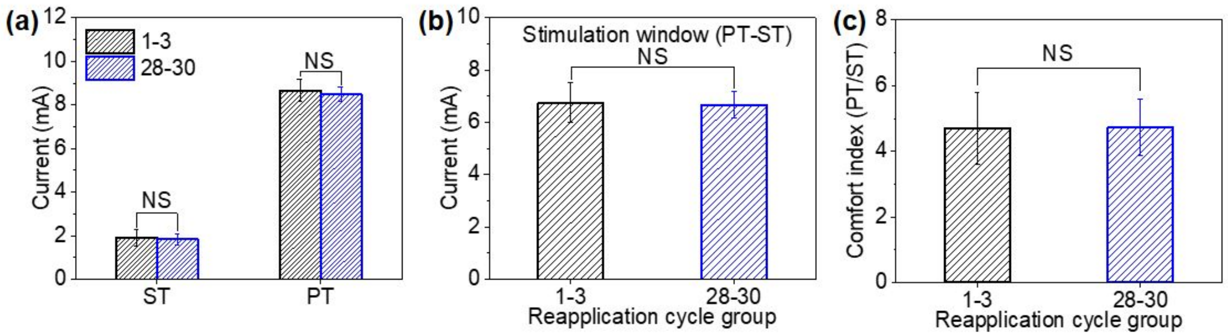

Figure S14. Electrical stimulation performance of nanocomposite electrodes (0.25% KCl) across repeated reapplication cycles. (a) Stimulation threshold (ST) and pain threshold (PT) currents for electrodes during early (cycles 1-3) and late (cycles 28-30) reapplication groups. **(b)** Stimulation window (PT-ST) for early and late cycles. **(c)** Comfort index (PT/ST) for early and late cycles. Error bars represent standard deviation (SD), and NS represents not significant (n = 3).

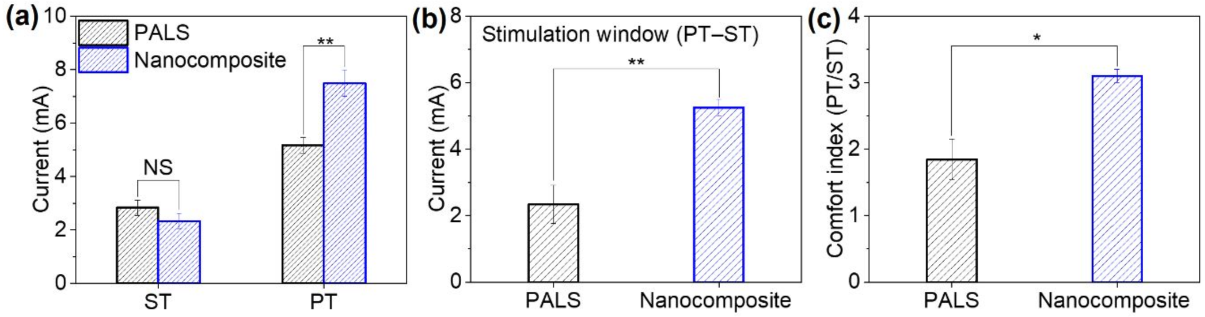

Figure S15. Stability of transcutaneous stimulation with reused PALS gel and nanocomposite electrodes. (**a**) Sensation threshold (ST) and pain threshold (PT) currents for PALS gel and nanocomposite electrodes. $P = 0.10119$ for comparison of the sensation threshold. $P = 0.00483$ for comparison of the pain threshold. (**b**) Stimulation window (PT-ST) for PALS gel and nanocomposite electrodes. $P = 0.00568$ ($n = 3$). (**c**) Comfort index (PT/ST) for PALS gel and nanocomposite electrodes. $P = 0.01285$ ($n = 3$). All error bars denote the standard deviation, and NS represents not significant. $^{*}P < 0.05$, $^{**}P < 0.01$.
